# Hyperphagia, Growth, and Puberty in Children with Angelman Syndrome

**DOI:** 10.3390/jcm12185981

**Published:** 2023-09-15

**Authors:** Karen G. C. B. Bindels-de Heus, Doesjka A Hagenaar, Ilonka Dekker, Danielle C. M. van der Kaay, Gerthe F. Kerkhof, ENCORE Expertise Center for AS, Ype Elgersma, Marie-Claire Y. de Wit, Sabine E. Mous, Henriette A. Moll

**Affiliations:** 1 Department of Pediatrics, Erasmus MC Sophia Children’s Hospital, University Medical Center Rotterdam, 3015 GD Rotterdam, The Netherlands; d.hagenaar@erasmusmc.nl (D.A.H.); i.dekker@erasmusmc.nl (I.D.); h.a.moll@erasmusmc.nl (H.A.M.); 2 ENCORE Expertise Center for Neurodevelopmental Disorders, Erasmus MC, University Medical Center Rotterdam, 3015 GD Rotterdam, The Netherlandsm.c.y.dewit@erasmusmc.nl (M.-C.Y.d.W.); s.mous@erasmusmc.nl (S.E.M.); 3 Department of Child- and Adolescent Psychiatry and Psychology, Erasmus MC Sophia Children’s Hospital, University Medical Center Rotterdam, 3015 GD Rotterdam, The Netherlands; 4 Department of Pediatric Endocrinology, Erasmus MC Sophia Children’s Hospital, University Medical Center Rotterdam, 3015 GD Rotterdam, The Netherlands; d.vanderkaay@erasmusmc.nl (D.C.M.v.d.K.); g.kerkhof@erasmusmc.nl (G.F.K.); 5 ENCORE Expertise Center for Neurodevelopmental Disorders, University Medical Center Rotterdam, 3015 GD Rotterdam, The Netherlands; 6 Department of Clinical Genetics, Erasmus MC, University Medical Center Rotterdam, 3015 GD Rotterdam, The Netherlands; 7 Department of Neurology and Pediatric Neurology, Erasmus MC Sophia Children’s Hospital, University Medical Center Rotterdam, 3015 GD Rotterdam, The Netherlands

**Keywords:** Angelman Syndrome, hyperphagia, height, BMI, puberty, genotype, longitudinal growth

## Abstract

Angelman Syndrome (AS) is a rare genetic disorder caused by lack of maternal UBE3A protein due to a deletion of the chromosome 15q11.2-q13 region, uniparental paternal disomy, imprinting center defect, or pathogenic variant in the *UBE3A* gene. Characteristics are developmental delay, epilepsy, behavioral, and sleep problems. There is some evidence for hyperphagia, shorter stature, and higher BMI compared to neurotypical children, but longitudinal studies on growth are lacking. In this study, we analyzed prospectively collected data of 145 children with AS, who visited the ENCORE Expertise Center between 2010 and 2021, with a total of 853 visits. Children showed an elevated mean score of 25 on the Dykens Hyperphagia questionnaire (range 11–55) without genotype association. Higher scores were significantly associated with higher body mass index (BMI) standard deviation scores (SDS) (*p* = 0.004). Mean height was −1.2 SDS (SD 1.3), mean BMI-SDS was 0.6 (SD 1.7); 43% had a BMI-SDS > 1 and 20% had a BMI-SDS > 2. Higher BMI-SDS was significantly associated with non-deletion genotype (*p* = 0.037) and walking independently (*p* = 0.023). Height SDS decreased significantly with age (*p* < 0.001) and BMI-SDS increased significantly with age (*p* < 0.001. Onset of puberty was normal. In conclusion, children with AS showed moderate hyperphagia, lower height SDS, and higher BMI-SDS compared to norm data, with increasing deviation from the norm with age. It is uncertain how loss of maternal UBE3A function may influence growth. Attention to diet, exercise, and hyperphagia from an early age is recommended to prevent obesity and associated health problems.

## 1. Introduction

Angelman Syndrome (AS) is a rare genetic disorder characterized by severe developmental delay, lack of speech, epilepsy, sleep and behavioral problems, and feeding issues [1,2]. The estimated prevalence of AS is 1:24,000 people [3]. AS is caused by loss of function of the maternally inherited *UBE3A* gene. The paternal *UBE3A* gene on chromosome 15q11.2q13.1 is silenced in neurons by imprinting. AS can occur due to a microdeletion of the 15q11.2-q13 region, a pathogenic variant of the *UBE3A* gene, a paternal uniparental disomy (UPD), or an imprinting center defect (ICD) [4,5]. The last three subtypes are more rare and generally have a less severe phenotype than children with a deletion. They are often analyzed as a “non-deletion group” [2,6,7].

In 2010, the national multidisciplinary ENCORE Expertise Center for AS was established at the Erasmus MC Sophia Children’s Hospital in Rotterdam, The Netherlands. A prospective study on natural history and genotype-phenotype differences of the first 100 children of this cohort showed a mean lower height and a mean higher weight for height (WfH) compared to norm data. Additionally, weight for height standard deviation scores (SDS) was significantly higher in the non-deletion group compared to the deletion children. In the non-deletion group, WfH SDS was higher in UPD/ICD children than in the children with a pathogenic variant in the *UBE3A* gene (*p* = 0.05) [2]. Similar findings in children with a UPD/ICD genotype have been reported before [6,7,8,9]. Parents reported hyperphagia in a third of the children, both in deletion and non-deletion subtypes [2]. Earlier small studies also report hyperphagia, specifically linked to the UPD genotype [9,10]. In our previous study, we did not report on birthweight, puberty, and target height [2]. Earlier reports show that children with UPD/ICD have a higher birth weight and suggest this to be a manifestation of intrinsic growth factors [7,10]. In our clinic, we see that the age of puberty onset is normal in girls and sometimes delayed in boys, but no study on the age of onset of puberty has been published. We are interested in the effect of mobility on body weight, expecting a positive association. Children with a deletion often have more feeding problems in infancy, and we want to investigate what the change in body weight is over time, as we have the impression that they go from a low BMI to a high BMI in childhood. We could not find any longitudinal studies on growth in AS.

Hyperphagia has a big social impact and is related to significant health risks [11,12]. Being overweight or obese comes with risks of serious comorbidity like type 2 diabetes mellitus, fatty liver disease, sleep apnea, and cardiovascular disease [13]. People with intellectual disability are at risk of developing obesity, and more knowledge on these aspects in AS is important to optimize prevention and treatments. Our cohort was expanded with five years of inclusion and follow-up, allowing for longitudinal analyses. We included data on birth weight, target height, pubertal development, and the ability to walk independently, and we assessed hyperphagia more objectively using a standardized questionnaire. The aim of this study was to gain more insight in hyperphagia, longitudinal growth and puberty, and associated factors in children with AS.

## 2. Materials and Methods

### 2.1. Study Design

This observational study presents prospectively collected data on eating behavior, growth, puberty, and possibly associated factors of 145 children with genetically confirmed AS between 0 and 18 years of age, who visited the ENCORE Expertise Center for AS at the Erasmus MC Sophia Children’s Hospital in Rotterdam, the Netherlands between April 2010 and December 2021. We excluded five children with a mosaicism for this study. Written informed consent was given by the parents of the children and permission to use the data for research purposes was given by the Medical Ethical Commission Erasmus MC Rotterdam (MEC-2015-203).

### 2.2. Data Collection

Data on growth, puberty, and possibly associated factors including epilepsy, use of anti-seizure medication (ASM), the ability to walk, scoliosis, and crouch gait were collected by the multidisciplinary team during the annual visit at the ENCORE Expertise Center for AS. Growth parameters, including height, weight, body mass index (BMI), target height, and birth weight were collected, and standard deviation scores (SDS) based on norm data of Dutch children were calculated [14]. Birth weight SDS was adjusted for gestational age [14]. Bone age was assessed at the age of 4, 7, 11, 15, and 18 years. BoneXpert software (version 3.0.3, Visiana, Holte, Denmark) was used for automated bone age estimation from a digital radiograph of the left hand [15].

Data on stages of puberty were categorized into three groups: (1) early puberty defined as B2 before 8 years and/or menarche before 10 years in girls and G2 and/or testicular volume of ≥4 mL before 9 years in boys, (2) normal puberty defined as B2 between 8–13 years and/or menarche between 10–14 years in girls, and G2 and/or testicular volume ≥ 4 mL between 9–13 years old in boys and (3) late puberty defined as B1 in girls of 13 years and older and/or menarche at 15 years and older, and G1 and/or testicular volume ≤ 4 mL in boys of 14 years and older. The association of growth with sex, genotype, epilepsy, use of ASM, and ability to walk independently was analyzed.

Hyperphagia was assessed by use of the Dutch version of the Dykens questionnaire, a parent reported questionnaire, originally designed for assessment of hyperphagia in Prader–Willi syndrome [12]. It contains 11 items with a range of 1 (normal) to 5 (severe). In addition to a total score, with a range from 11 (no hyperphagia) to 55 (severe hyperphagia), it also gives subscores on drive, behavior, and severity. The total score has no pathological cut-off. It was sent to parents with the invitation for their annual visit in 2021. Questionnaires with five or more missing answers were excluded. Single missing answers were imputed with the mean of the group on that specific item.

### 2.3. Statistical Analysis

Individuals with a UPD, ICD, or a pathogenic variant in the *UBE3A* gene who do not have a deletion of the 10 non-imprinted genes in the 15q11.2-q13 locus were combined in a “non-deletion” group and compared with children of the “deletion” group for the cross-sectional analyses. We also analyzed UPD and ICD children together because of their similar genotypes and compared them to children with a pathogenic variant in the *UBE3A* gene [2,6,7].

Cross-sectional statistical analyses using data of the most recent visit were performed in IBM SPSS Statistics Data Editor version 25 [16]. The differences between groups were calculated with unpaired *T*-tests for continuous, normally distributed data, and with Chi-square tests for categorical data. Not normally distributed numerical data were analyzed with a Mann–Whitney U test. Differences of the AS children from the neurotypical Dutch population were tested using a One Sample T-test. To look for factors that were associated with hyperphagia, growth, and puberty, multiple linear regression analysis was used while controlling for sex and age. *p*-values < 0.05 were considered statistically significant and displayed with its effect size.

Longitudinal data were analyzed in R version 4.0.5 [17]. Two mixed effects models [18] were conducted for the outcome variables height SDS and BMI-SDS, with age as predictor. Covariates were sex and genotype. Genotype was categorized into deletion, UPD, ICD, and *UBE3A* mutation, as the mixed effects model allows for the use of smaller groups. Independent walking (yes/no) and epilepsy (yes/no) were added as covariate if they showed a significant correlation with the outcome at most recent visit (Spearman’s ρ). Likelihood Ratio (LR) Tests and T-tests were used to obtain *p*-values, while the Bayesian information criterion (BIC) and Akaike Information Criterion (AIC) were used as additional informants of model quality.

## 3. Results

### 3.1. Patient Characteristics at Most Recent Visit

Table 1 presents the characteristics of 145 children with AS. Of the 145 children, 114 (79%) visited the center two or more times and 83 (57%) five or more times.

Mean birth weight was −0.6 SDS. Children with a UPD/ICD genotype had a significantly higher mean birth weight than children in the *UBE3A* group (*p* = 0.002, Cohen’s *d* = 1.085). Children with a deletion had a significantly higher prevalence of epilepsy (*p* < 0.001, Cramers V = 0.332), ASM use (*p* < 0.001, Cramers V = 0.348), and less children walked independently (*p* < 0.001, Cramers V = 0.521).

### 3.2. Hyperphagia

In total, 58 (71%) out of 82 questionnaires were returned by the biological parents (5 were excluded due to comorbidity or 5 missing items or more). The results of 53 questionnaires are displayed in Table 2. Mean scores per item are reported in Table A1 in the Appendix A.

There was no significant association between total and subscale scores on the Dykens questionnaire and age, sex, or genotype (deletion versus non-deletion and UPD-ICD versus mutation). There was a significant association between the Dykens total score and BMI-SDS, corrected for age, sex, and genotype (*p* = 0.004).

### 3.3. Growth: Cross-Sectional Results

Growth data from the most recent visit are displayed in Table 3.

**Figure 1:**
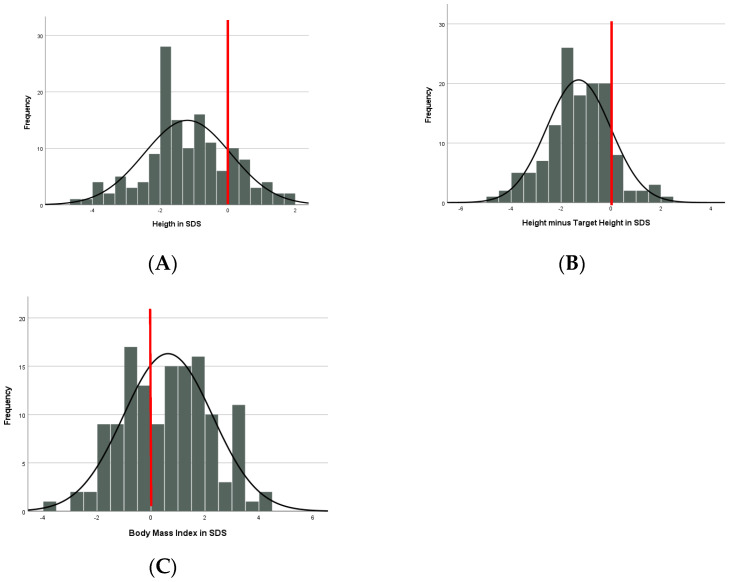
(**A**) Height (in SDS). (**B**) Height minus Target Height (in SDS). (**C**) BMI (in SDS). The red line displays the mean line of neurotypical children.

The mean height was −1.19 SDS without sex or genotype difference (Table 3, Figure 1A). Children with AS were shorter than expected from Dutch references (*p* < 0.001) and their target height SDS (*p* < 0.001). Mean height was lower than expected from the target height, but within the normal range of ±1.6 SDS [14] (Table 3, Figure 1B). In 42 children with available bone age, mean bone age was close to the calendar age (−0.31 SDS (SD = 1.4)) without differences between the genotype subgroups.

Mean BMI was 0.63 SDS (Table 3, Figure 1C) without sex difference. Children in the non-deletion group had a significantly higher BMI-SDS compared to the deletion group, corrected for age (*p* = 0.037, Partial Eta squared 0.032). There was no difference in BMI-SDS within the non-deletion group, corrected for age (*p* = 0.332). Fortythree percent of all children had a BMI > 1 SDS with a significantly higher proportion in the non-deletion group (*p* < 0.001, Cramers V = 0.318); 20% had obesity (BMI > 2 SDS), without genotype difference.

There was no association between height and scoliosis or crouch gait. The use of valproic acid was associated with a lower height (*p* = 0.048), but this disappeared when corrected for age and genotype (*p* = 0.145).

A significant association was found between a higher BMI-SDS and independent walking (*p* < 0.001, Cohen’s *d* = −0.637); this persisted when corrected for age and genotype (*p* = 0.023, standardized beta 0.245). When only analyzing the group of children older than two years of age, when neurotypical children are expected to walk independently, the association persisted (*p* = 0.003, Cohen’s *d* = −0.570); also when corrected for age and genotype (*p* = 0.034, standardized beta = 0.229).

### 3.4. Growth: Longitudinal Results

Of the 145 children, 114 had 2 or more height measurements, and on average 4–5 measurements per child were available. Data of 822 height measurements were analyzed.

**Figure 2:**
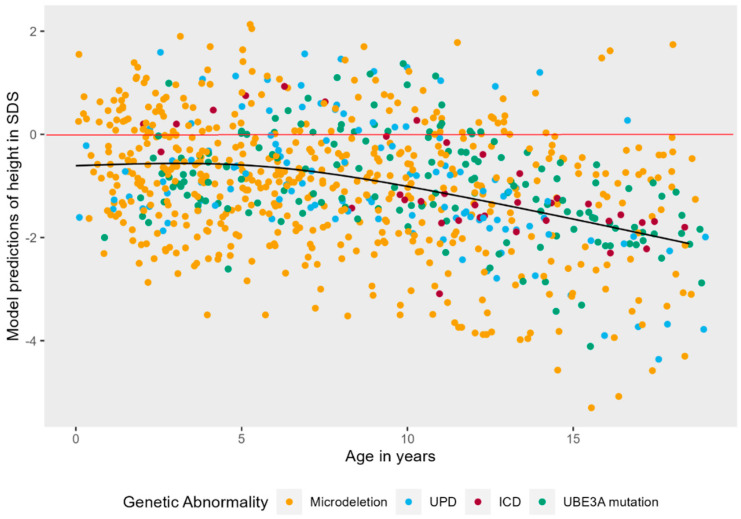
Non-linear mixed effects models displaying the longitudinal course of height in SDS in AS patients. The black line represents the predicted values of height in SDS for AS patients over time, while the red line represents the mean height in SDS of neurotypically developing children.

There was no significant correlation between height and epilepsy (ρ = −0.102) or independent walking (ρ = 0.164). The model with the highest fit to the data was a model with a non-linear natural cubic splines effect of age (3 knots), random intercepts, and non-linear random slopes. Figure 2 shows that there was a significant main effect of age on height in SDS, LR = 51.10, *p* < 0.001. As children with AS become older, the deviation from normal becomes larger. This effect is non-linear: height in SDS stays stable from 0 to approximately 5 years, and then starts to decrease. No main effect of genotype or sex was detected.

**Figure 3:**
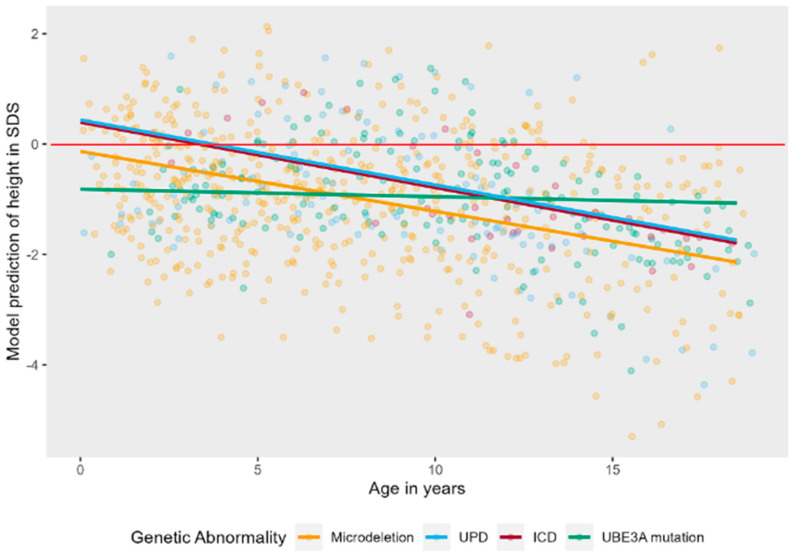
Linear mixed effects models displaying the longitudinal course of height in SDS in AS patients per genotype. The red line represents the mean height in SDS of typically developing children.

**Figure A1:**
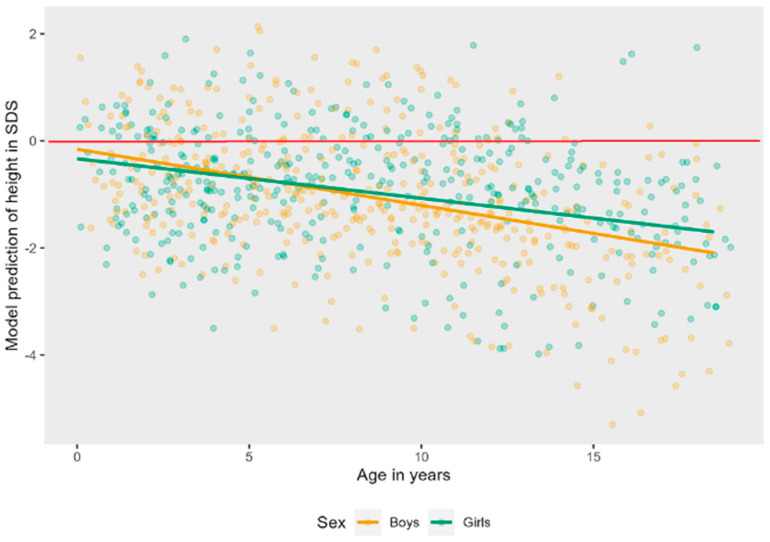
Linear mixed effects models displaying the longitudinal course of height in SDS in AS patients per sex. The red line represents the mean height in SDS of typically developing children.

The interaction effect of age and genotype was tested on a linear model to ease interpretation. There was a significant interaction effect, LR = 10.64, *p* = 0.014, see Figure 3. Post hoc tests showed that the trajectory of height in SDS over time is significantly different in patients with a UBE3A mutation as compared to deletion (B = 0.09, t (821) = 3.17, *p* = 0.002) and UBE3A mutation as compared to UPD (B = 0.10, t (821) = 2.57, *p* = 0.010). Height SDS stays stable over time for patients with a UBE3A mutation, while it decreases in the other groups. There is no significant interaction of age and sex on height SDS, LR = 1.66, *p* = 0.197. Boys and girls do not differ in their trajectory of height SDS over time (see also Figure A1 in Appendix A).

**Figure 4:**
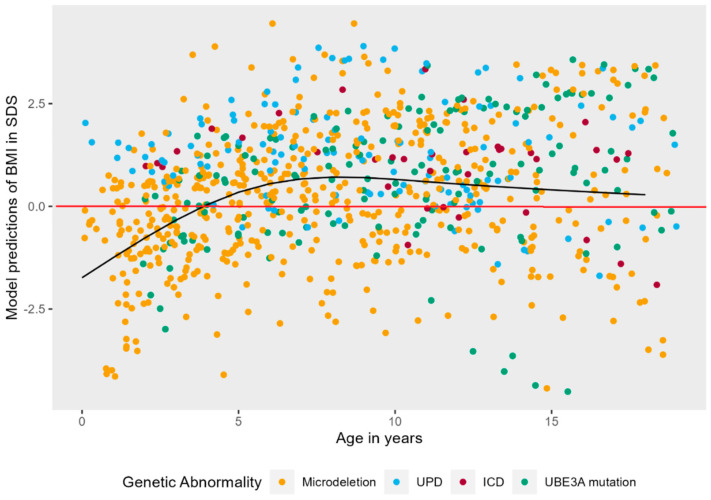
Non-linear mixed effects models displaying the longitudinal course of BMI in SDS in AS patients. The black line represents the predicted values of BMI in SDS for AS patients over time, while the red line represents the mean BMI in SDS of typically developing children.

For BMI, 853 measurements were analyzed. BMI-SDS was significantly correlated with independent walking (ρ = 0.265), but not with epilepsy (ρ = 0.157), thus independent walking was added as covariate. The model with the highest fit to the data was a model with a non-linear natural cubic splines effect of age (3 knots), random intercepts, and non-linear random slopes. As shown in Figure 4, there was a significant main effect of age on BMI-SDS, LR = 79.39, *p* < 0.001. Young children with AS typically have low BMI-SDS as compared to their peers. As children with AS become older, weight increases, and their BMI-SDS transcends the comparison line (in red) at approximately four years old, ending somewhat above zero at six years old, where it remains relatively stable.

**Figure A2:**
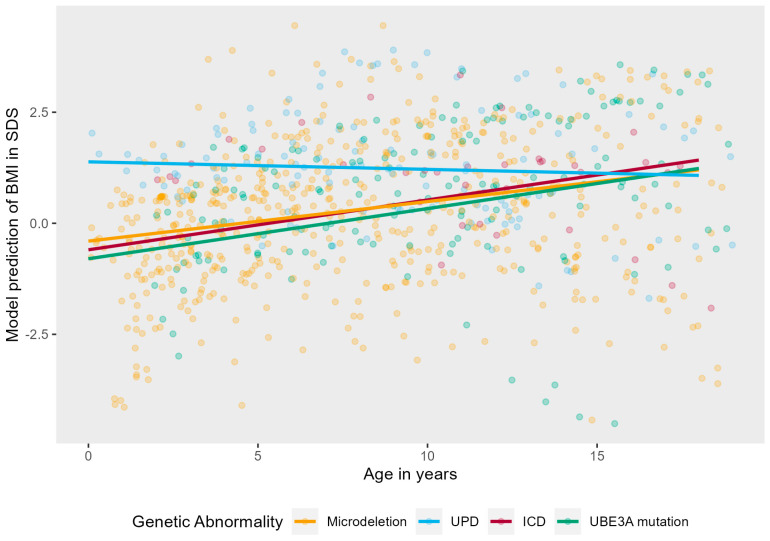
Linear mixed effects model displaying the longitudinal course of BMI in SDS in AS patients per genotype. The red line represents the mean BMI in SDS of typically developing children.

**Figure A3:**
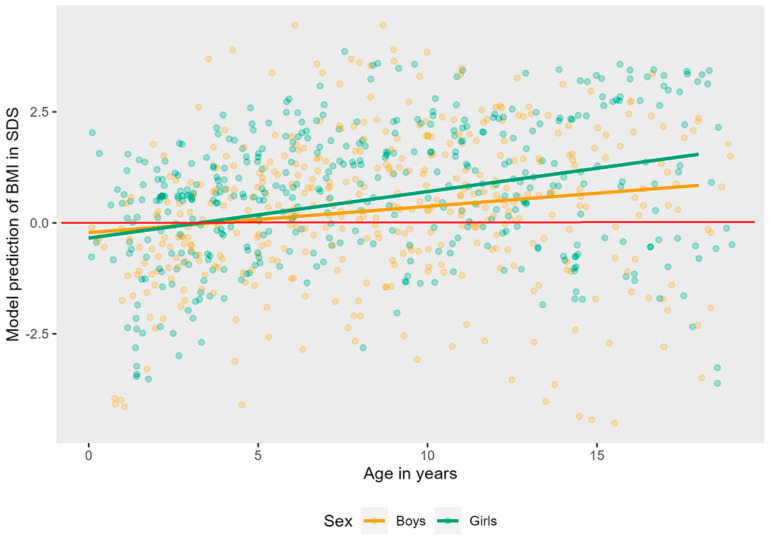
Linear mixed effects model displaying the longitudinal course of BMI in SDS in AS patients per sex. The red line represents the mean BMI in SDS of typically developing children.

**Figure A4:**
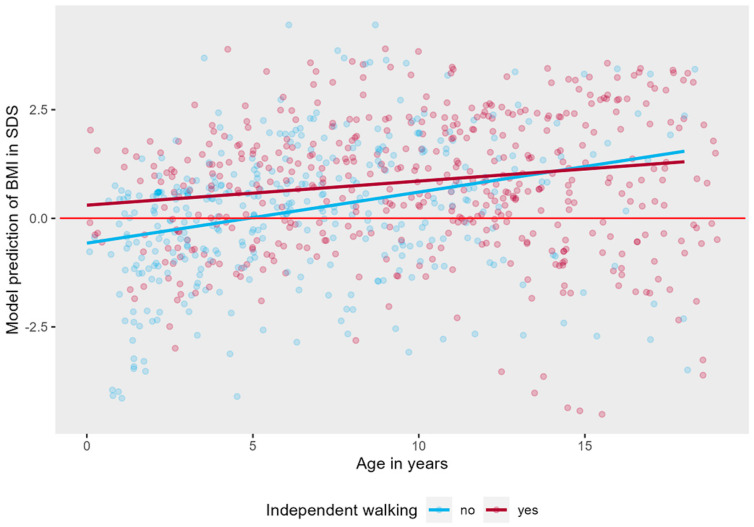
Linear mixed effects model displaying the longitudinal course of BMI in SDS in AS patients per walking group. The red line represents the mean BMI in SDS of typically developing children.

As can be seen in Figure A2, Figure A3 and Figure A4 in Appendix A, there was no significant interaction effect between age and genotype (LR = 4.08 *p* = 0.253), sex (LR = 1.31 *p* = 0.252), or independent walking (LR = 2.32, *p* = 0.127).

### 3.5. Puberty

There was no genotype difference in onset of puberty and age at menarche. Of 53 girls over the age of 8 years, 25 girls had their menarche at a mean age of 11.6 years (SD = 1.9 years) (Table A2 in the Appendix A). Age of first appearance of B2 was 11.5 (SD = 2.1) years (n = 13). In boys, mean age of appearance of G2 was 13 years (SD = 1.9 years, n = 15), while mean age of appearance of testicular volume of 4 mL was 13.5 years (SD = 1.3 years, n = 14). Two boys received testosterone treatment because of delayed puberty at the age of respectively 16 and 17 years old.

### 3.6. Tube Feeding

In contrast to hyperphagia, there were also children with feeding problems. Seventeen children (12%) of which 13 had a deletion and 4 a non-deletion, needed current (n = 12) or previous (n = 5) tube feeding. Reasons for chronic tube feeding were insufficient intake and/or swallowing disorders and for intercurrent tube feeding, status epilepticus, or feeding problems after surgery.

## 4. Discussion

In a large prospective clinical cohort of 145 children with AS, data on hyperphagia, growth and puberty were analyzed both cross-sectionally and longitudinally. Children with AS showed signs of hyperphagia on the Dykens questionnaire with an elevated total mean score of 25 (SD = 9.0) on a scale of 11–55. There was no association with genotype, sex, or age. Higher scores were significantly associated with a higher BMI-SDS. Children showed a significantly shorter stature compared to the reference population and their target height. There was no association of height with genotype cross-sectionally. Height SDS decreased significantly during childhood and adolescence. Children with AS had a higher BMI-SDS than neurotypical children; 43% of the children had a BMI ≥ 1 SDS and BMI-SDS was significantly higher in the non-deletion group. BMI-SDS increased significantly with age. Pubertal development was within the normal range.

### 4.1. Hyperphagia

Hyperphagia has been previously recognized in AS. Welham used a different food-related behavior problems questionnaire and found scores of patients with AS (n = 25) approaching and sometimes exceeding scores of patients with PWS [10]. Mertz used the Dykens questionnaire in 39 patients with AS, with total scores between 17.5 (deletion group) and 29.8 (UPD group). No association analyses with growth parameters were reported [9].

We compared our results with studies using the Dykens questionnaire in patients with other genetic disorders with known hyperphagia (Table A3 in the Appendix A) [11,12]. Mean scores of our AS population are approximately 2 points below the mean scores of PWS, Bardet Biedl Syndrome, and Alström Syndrome, and they are 6–7 points below the mean of children with defects in the leptin–melanocortin pathway, regulating hunger and satiety (Table A3 in the Appendix A). Children with AS have a lower developmental level (including poorer communication and motor skills) than children with PWS [19], and it is therefore possible that the total score is influenced by the fact that children with AS are not able to independently acquire food. The finding in our study of a higher BMI-SDS in children that walk independently supports this hypothesis.

Appetite, satiety, and energy expenditure are regulated by a complex neuro-endocrine interplay between neuronal signals from the hypothalamus, anorexigenic (leptin) and orexigenic (ghrelin) hormones, gastro-intestinal hormonal and stretching signals, and environmental stimuli (vision and smell) [20,21,22,23]. An intact leptin–melanocortin pathway is essential for regulation of satiety, which, for example, can be disturbed by LEPR and MC4R mutations [11]. It is yet unknown whether one or more of these pathways are also involved in AS. One possible factor in increased hyperphagia in AS is the association between *UBE3A* gene and circadian rhythm dysfunction, which can lead to sleep problems [24]. Sleep disorders are a well-known phenomenon in AS [1,2,7]. Mouse model research suggests that eating behavior problems are related to disturbances in the circadian rhythm, and/or that a disturbed sleep cycle leads to obesity [25] In humans, working a night shift has an effect on appetite and weight [21]. This possible relationship would be another reason for sleep evaluation during clinical visits and consider pharmacological and/or behavioral interventions [26].

UBE3A has been shown to be involved in nuclear hormone receptor functioning in the mouse brain [27]. This was especially noted in relation with the responsiveness to acute changes in corticosteroid levels. UBE3A may also be involved in other hormone axis regulation. Since we saw no genotype influence in hyperphagia and BMI, we feel that the earlier hypothesis of increased appetite and growth in UPD due to paternal overexpression of the *MAGEL2* gene to be less likely [9]. Other factors to consider would be different manifestations of sensory disorders, e.g., mouthing behavior and oral hyporesponsiveness, which are often seen in AS [28]. Also, intellectual disability in itself could explain impaired behavioral regulation. Children with AS have low scores on IQ tests [2,7]. The cognitive functioning scores of our patients were not evaluated at approximately the same time as the hyperphagia assessment, so we could not analyze a possible association.

We do not have longitudinal data of the hyperphagia questionnaire, which would be needed to investigate whether there are specific patterns over time, as is known in PWS [29]. Hyperphagia is a problem with a large social impact on the child and its family. Having a BMI higher than the healthy weight range has negative consequences for their longterm health. Further research is needed on the underlying mechanisms and possible interventions.

### 4.2. Growth

We found that mean birth weight SDS (corrected for gestational age) was within the normal range for all genotypes. Earlier studies have shown a higher birth weight in children with UPD and hypothesized an association with overexpression of paternally expressed genes [9]. Our cohort also showed a significantly higher birth weight SDS for non-deletion children compared to the deletion children, but this effect was driven by the five children with an ICD, not UPD (1.03 versus −0.22 SDS). We do not feel that conclusions can be drawn based on this low number of children in the ICD group. Our data suggest that the growth problems in AS are a postnatal process.

Children with AS have a mean height of −1.2 SDS below the mean of neurotypical children. There was no physical explanation, such as scoliosis, crouch gait, or delay in bone age. Longitudinal analyses showed that from approximately 5 years of age onwards, height SDS decreased with no catch-up growth in puberty as would be expected, resulting in an adult height of approximately −2 SDS. We found a significant difference in course over time of height between children with a pathogenic variant in the *UBE3A* gene versus those with a deletion and UPD, with a lower height SDS starting from a young age, remaining more stable in the first group. We cannot think of an explanation for that. All groups show a comparable height at the end of adolescence. Valproic acid can negatively affect growth in rats [30], but we found no significant association between height and use of valproic acid. No studies on growth hormone secretion or thyroid function as possible causal factors of lower height are available.

Mean BMI-SDS is higher in AS than in neurotypical children, with a significant difference between deletion and non-deletion children. There were no significant differences between children with UPD, ICD, and pathogenic variants in the *UBE3A* gene. This contrasts with the study of Brennan (maximum age 44 months) and Tan (maximum age 60 months), who found children with UPD/ICD having the highest BMI in their AS cohorts [7,8]. Possibly, this is related to the observed growth pattern of children with UPD starting at a higher weight at young age, but staying stable over time, while we see an increasing BMI-SDS over time for the other three genotype subgroups, although this was not a statistically significant effect. The percentage of children in our study falling in the overweight range and with obesity was 23% and 20%, respectively. In 2021 in the general Dutch population, 11.2% of children fell into the overweight range and 2.5% were obese [31]. Longitudinal analysis showed a significant increase in BMI-SDS over time. To our surprise, a higher BMI-SDS was significantly associated with being able to walk independently. BMI is a crude parameter for body composition, it does not give information about percentage of fat versus muscle mass; children who walk could have more muscle mass. Another explanation could be that children who walk independently are able to acquire food by themselves.

We studied children with AS until 18 years of age. In a study of 100 adults with AS in the Netherlands, 25% percent of women and 22% of men were at least 2 SDS below the mean height of the Dutch population [32]. Analysis of BMI showed 37% of adults being overweight (BMI ≥ 25), which is comparable to our findings. However, adults did show a sex difference; 24% of men and almost 50% of women were overweight. Obesity was found in 9% of males and females, which is a lower prevalence than in children. Almost half of the caregivers reported that their (adult) child has difficulties with portion control or waiting for food and reported the use of diets to maintain a healthy weight. The authors suspect that more awareness of the importance of a healthy diet could lead to a healthier weight in adulthood, and we recommend monitoring of weight and intake and appropriate support in children and adults with AS to prevent obesity [32].

As stated before, UBE3A has been implicated in the circadian rhythm regulation [24,33] which can affect appetite and growth [21,25]. In mice, increasing neuronal CAMK2 signaling led to reversal of the overweight phenotype of *Ube3a* mice, supporting the hypothesis of its neuronal origin [34]. Furthermore, children with PWS have a lower resting energy expenditure, and this may also be true for children with AS [35]. Increase in caloric intake can result in rapid weight gain, most of which is presumed to be fat mass [36]. No studies on resting energy expenditure and body composition in AS are known.

We advise to support parents from early age to keep a healthy diet, to avoid using sweets as a treat or reward, and to have a dietician evaluate the diet as necessary.

### 4.3. Puberty

Objective information about onset of puberty was not published before, except from general comments about normal timing of puberty in reviews [37,38]. The mean age at menarche in our cohort was within the normal range, but it was relatively early compared to the mean age of 12–13 years in neurotypical children [39,40,41]. In boys, 10% showed a late onset of puberty. The normal range of age of onset of puberty in boys is wider than in girls with skewing to the right, but late puberty is seen in only 0.6–2.3% in the norm population [42,43].

### 4.4. Strengths and Limitations

An important strength of this study is the large clinical cohort of 145 children with AS with prospectively collected, detailed, and standardized data on a broad range of relevant aspects of this rare disorder. It also spans more than 10 years of follow-up, which enabled us to do longitudinal analyses. This led to new knowledge about growth in AS. Adding the target height gives more objective information about height of the children. Pubertal development was never reported this detailed.

This study also has some limitations. The Dykens questionnaire has no pathological score cut-off. It also assesses food behavior, not the actual food intake. We cannot be sure that the higher body weight is a consequence of a higher food intake or that both hyperphagia and a higher BMI-SDS are a manifestation of the same underlying molecular–genetic problem. Further research is needed to obtain more information on actual amount and type of food intake. We also do not have information on body composition and energy expenditure. We analyzed the association of the ability to walk with weight but were not informed on how much children walk in daily life. The data on onset of puberty were partly by recall of the parents, as there was at least one year between two appointments. Furthermore, even with 145 children and 10 years of follow-up, there are still small numbers in the ICD group.

## 5. Conclusions

Children with AS show moderate signs of hyperphagia, with a significant association of hyperphagia with BMI-SDS. Furthermore, children with AS have a shorter stature than neurotypical children and their height SDS significantly decreases with age. They have a higher BMI-SDS than children without AS and this increases significantly with age. Children in the non-deletion group have a significantly higher BMI-SDS than children in the deletion group. Pubertal development is normal. Further research is necessary to explore the resting energy expenditure, body composition, and hormone levels associated with appetite and growth in AS and the relation to the loss of the *UBE3A* gene and other genes in that region. More insight in underlying mechanisms could lead to better options for intervention. We recommend monitoring growth in children with AS and informing and advising parents on diet, exercise, and how to manage hyperphagia to prevent obesity and associated comorbidity.

## Figures and Tables

**Table 1:** General characteristics at most recent visit of 145 children with AS.

Genotype	Deletion	Non-Deletion	Mean/Total
N (% of total group = 145)	89 (61)	56 (39)	145
*UPD* (% of total group)		*18 (12)*	
*ICD* (% of total group)		*5 (3)*	
*UBE3A* (% of total group)		*33 (23)*	
Sex (m/f)	51/38	27/29	78/67
*UPD*		*8/10*	
*ICD*		*3/2*	
*UBE3A*		*16/17*	
Mean age years (SD)	9.7 (6.2)	11.6 (6.1)	10.5 (6.2)
Birth weight SDS (SD) **^#^	−0.46 (1.0)	−0.80 (1.4)	−0.59 (1.2)
*UPD*		*−0.22 (1.1)*	
*ICD*		*1.03 (0.8)*	
*UBE3A*		*−1.39 (1.2)*	
Epilepsy (N (% of subgroup)) *	77 (87)	33 (59)	110 (76)
ASM (N (% of subgroup)) *	75 (84)	31 (55)	104 (71)
*Valproic acid*	*36 (40)*	*12 (21)*	*48 (33)*
Independent walking			
(N (% of subgroup)) *	34 (38)	48 (86)	82 (57)

UPD = uniparental paternal disomy, ICD = imprinting center defect, ASM = anti-seizure medication. * significant difference between deletion and non-deletion (*p* < 0.001). ** significant difference between UPD/ICD and *UBE3A* (*p* = 0.002). ^#^ birthweight based on 101 cases.

**Table 2:** Hyperphagia score based on Dykens questionnaire.

Genotype	Deletion (n = 31)	Non-Deletion (n = 22)	Total Group (n = 53)
Total score (SD) ^#^ (range 11–55)	25.3 (9.6)	24.8 (8.2)	25.1 (9.0)
*UPD-ICD*		*26.3 (8.6)*	
*UBE3A*		*23.5 (8.0)*	
Subscale behavior (SD) (range 5–25)	11.6 (4.8)	11.4 (4.0)	11.5 (4.4)
Subscale drive (SD) (range 4–20)	10.5 (4.0)	9.8 (3.7)	10.2 (3.9)
Subscale severity (SD) (range 2–10)	3.3 (1.9)	3.6 (1.7)	3.4 (1.8)

UPD = uniparental paternal disomy, ICD = imprinting center defect. ^#^ 8 missing items for 6 children in total (item 2, 5, 6, 8 and 9, see Table A1 in the Appendix A).

**Table 3:** Growth and puberty parameters at most recent visit.

Genotype	Deletion	Non-Deletion	Total Group	N
Height SDS (SD)	−1.23 (1.4)	−1.13 (1.1)	−1.19 (1.3)	144
*UPD*		*−1.18 (1.4)*		
*ICD*		*−1.24 (1.0)*		
*UBE3A*		*−1.07 (1.0)*		
BMI-SDS (SD) *	0.37 (1.7)	1.03 (1.5)	0.63 (1.7)	135
*UPD*		*1.43 (1.4)*		
*ICD*		*0.83 (1.7)*		
*UBE3A*		*0.82 (1.5)*		
BMI ≥ 1 SDS (N (% of subgroup)) *	25 (30)	33 (62)	58 (43)	
Overweight (BMI ≥ 1–<2 SDS) (N (% of subgroup)) *	10 (12)	21 (40)	31 (23)	
Obesity (BMI ≥ 2 SDS) (N (% of subgroup))	15 (18)	12 (22)	27 (20)	135
Target height SDS (SD)	0.20 (0.1)	0.06 (0.1)	0.07 (0.87)	133
Height—Target height SDS (SD)	−1.32 (1.4)	−1.26 (1.1)	−1.30 (1.3)	133
Age at menarche in years (SD)	11.8 (2.4)	11.4 (1.2)	11.6 (1.9)	25
Onset of puberty (N (% of subgroup))				
*Prepubertal*	41 (46)	18 (32)	59 (41)	
*Early*	3 (3)	0	3 (2)	
*Normal*	40 (45)	31 (55)	71 (49)	
*Late*	5 (6)	7 (13)	12 (8)	145

UPD = uniparental paternal disomy, ICD = imprinting center defect. * Significant difference between deletion and non-deletion (BMI-SDS *p* = 0.037, BMI-SDS > 1 *p* < 0.001, overweight *p* < 0.001).

## Data Availability

The data presented in this study are available on reasonable request from the corresponding author.

## Appendix A

**Table A1:** Dykens questionnaire results per item ^#^.

	N	Mean	SD
1. How upset does your child generally become when denied a desired food?	53	2.75	1.21
2. How often does your child try to bargain or manipulate to get more food at meals?	53	3.19	1.70
3. Once your child has food on their mind, how easy is it for you or others to re-direct your child away from food to other things?	53	2.96	1.34
4. How often does your child forage through the trash for food?	53	1.30	0.91
5. How often does your child get up at night to food seek?	53	1.10	0.49
6. How persistent is your child in asking or looking for food after being told “no” or “no more”?	53	2.63	1.22
7. Outside of normal meal times, how much time does your child spend talking about food or engaged in food-related behaviors?	53	1.75	1.13
8. How often does your child try to steal food (that you are aware of?)	53	2.76	1.64
9. When others try to stop your child from talking about food or engaging in food-related behaviors, does it leads to distress or being upset?	53	1.81	1.11
10. How clever or fast is your child in obtaining food?	53	3.13	1.39
11. To what extent to food-related thoughts, talk, or behavior interfere with your child’s normal daily routines, self-care, school, or work?	53	1.64	0.94

^#^ Subscores calculation: Drive: item 1 + 3 + 6 + 9 (range 4–20); Severity: item 7 + 11 (range 2–10) and Behavior: item 2 + 4 + 5 + 8 + 10 (range 5–25).

**Table A2:** Onset of puberty in AS.

	Girls (N (%))	Boys (N (%))	Total (N (%))
Prepubertal	27 (40.3)	32 (41.0)	59 (40.7)
Early	3 (4.5)	-	3 (2.1)
Normal	33 (49.2)	38 (48.7)	71 (49.0)
Late	4 (6.0)	8 (10.3)	12 (8.3)
Total	67	78	145

**Table A3:** Dykens scores in different populations.

	Total Score (11–55)	Behavior (5–25)	Drive (4–20)	Severity (2–10)
AS (this study) N = 53	25.1 (9.0)	11.5 (4.4)	10.2 (3.9)	3.4 (1.8)
AS (Mertz, 2014 [9]) N = 39 ^#^	21.8	10.0	8.7	3.1
PWS (Dykens, 2007 [12]) N = 153	27.9	13.6 (4.5)	12.3 (3.3)	4.6 (1.6)
PWS (Zorn, 2022 [11]) N = 649 *	27.0			
BBS (Zorn, 2022 [11]) N= 13	27.6 (9.0)	12.5 (4.1)	11.2 (4.1)	3.8 (1.5)
LEPR (Zorn, 2022 [11]) N = 8	32.0 (9.3)	13.4 (3.9)		
MC4R (Zorn, 2022 [11]) N = 7	31.4 (5.4)	11.9 (2.3)		
Alström (Zorn, 2022 [11]) N = 23	27 (13)	-	-	-
16p11.2del (Zorn, 2022 [11]) N = 5	21.4 (5.5)	8.8 (2.2)	10.0 (3.1)	2.6 (0.9)

BBS = Bardet Biedl Syndrome, LEPR = leptin receptor, MC4R = melanocortin-4-receptor. ^#^ scores calculated from data reported in Mertz et al. [9], based on the mean of the subscores per genotype. * scores calculated from data reported in Zorn et al. [11], based on the mean of 17 studies.
